# Myocardial Mechanics and Valvular and Vascular Abnormalities in Hemophilia

**DOI:** 10.31083/RCM39387

**Published:** 2025-09-28

**Authors:** Attila Nemes

**Affiliations:** ^1^Department of Medicine, Albert Szent-Györgyi Medical School, University of Szeged, H-6725 Szeged, Hungary

**Keywords:** myocardial, vascular, valvular, hemophilia

## Abstract

Hemophilia is an X-linked pathology characterized by a deficiency or lack of certain coagulation factors. This review aims to summarize the present literature describing the abnormalities in myocardial, valvular, and vascular morphology and function associated with hemophilia. While the present findings are limited, recent developments in cardiovascular imaging foreshadow the possibility that future research on the topic will continue to expand.

## 1. Hemophilia

Hemophilia is considered an X-chromosome-linked recessive inheritance disorder characterized by a deficiency or lack of certain coagulation factors, mostly affecting males. Women are usually heterozygous carriers of the mutated gene and present with only mild symptoms. Currently, only deficiencies in coagulation factors VIII (hemophilia A) and IX (hemophilia B) are considered to promote hemophilia. Other types of clotting factor deficiencies, such as factor XI (hemophilia C in the past), are regarded as rare bleeding disorders. Both hemophilia A and B have similar symptoms, such as different forms of bleeding (for instance, hemarthrosis causes hemophilic arthropathy); the severity of the disease is associated with the number of residual factors. A definite diagnosis is performed by measuring residual coagulation factor activity. Due to the availability of safe blood coagulation factor concentrates and other treatment options, life expectancy for patients with hemophilia has improved significantly in the last decades. Therefore, owing to the increased life expectancy of patients, the question arose as to what other subclinical abnormalities are associated with the disease [1, 2].

In hemophilia, atherosclerosis develops at similar rates to those in the general population; however, the incidence of atherothrombosis and cardiovascular disease-related mortality is much lower, due to the hypocoagulable state present in hemophilia patients [3, 4, 5]. Results from a prospective population-based study found that the extent of coronary artery atherosclerosis is comparable between older men with and without hemophilia, suggesting the importance of screening and treating atherosclerosis risk factors in hemophilia patients [6]. Clinically diagnosed hypertension, insulin resistance, and hyperlipidemia were also shown to be more prevalent in adults with moderate and severe hemophilia compared to controls [7]. Therefore, active identification of classic cardiovascular risk factors (obesity, hypertension, hypercholesterolemia, etc.) is required, since these risk factors may be responsible for the development of cardiovascular abnormalities associated with the disease. Moreover, balancing between bleeding risk and thrombotic management is essential in this disorder [8]. Most papers assessing cardiovascular abnormalities in hemophilia report on the management of a hemophilic patient during a possible surgery [9], the treatment of hemophilic patients with prosthetic valves [10], or the management of patients with co-existing peripheral [11] or coronary artery disease or atrial fibrillation [12]. Therefore, this review aimed to summarize the current literature regarding abnormalities in myocardial, valvular, and vascular morphology and function associated with hemophilia. While the findings are limited, recent developments in cardiovascular imaging foreshadow the possibility that future research on the topic will continue to expand. Case reports of single cases and reports of patients with co-existing hemophilia and other disorders have not been involved, only papers on series of hemophilia subjects. Nonetheless, the presence of ageing-related abnormalities cannot be excluded.

## 2. Cardiovascular Imaging and Magyar-Path Study

Cardiovascular imaging has undergone tremendous technical advances in recent decades, and in addition to echocardiography, which had a previous dominance, cardiac computer tomography and magnetic resonance imaging have also appeared. New cardiac ultrasound procedures have also emerged, such as speckle-tracking echocardiography (STE) and three-dimensional (3D) echocardiography, and their combination (3DSTE). One of the newest echocardiographic developments, 3DSTE enables detailed assessment of dimensions and functional features not only of the heart chambers, but the valves as well, using virtually acquired 3D echocardiographic datasets [13, 14, 15, 16, 17]. In the present summary, all results regarding hemophilia-associated myocardial, valvular, and vascular abnormalities demonstrated are from our own “Motion Analysis of the heart and Great vessels bY three-dimension Al speckle-tRacking echocardiography in Pathological cases” (MAGYAR-Path) Study [18, 19, 20, 21]. When conducting 3DSTE, all hemophilia patients being cared for and treated at the tertiary Hematology Center of the University of Szeged were involved in the study. For comparisons, healthy subjects served as controls without any known states that could affect findings. A Toshiba Artida® echocardiography tool (Toshiba Medical Systems, Tokyo, Japan) was used for two-dimensional (2D) Doppler echocardiography (attached to a PST-30BT transducer) and 3DSTE (attached to a PST-25SX) in all cases. The 3DSTE-derived echocardiographic datasets were analyzed using a 3D Wall Motion Tracking software (version 2.7, UltraExtend, Toshiba Medical Systems, Tokyo, Japan). This study proposed to demonstrate the diagnostic and prognostic impact of 3DSTE-derived parameters in certain pathologies, such as hemophilia, since 2011. Only abnormalities related to the large arteries were listed from vascular parameters.

## 3. The Left Heart and the Aorta

### 3.1 Left Ventricle

#### 3.1.1 In Healthy Subjects 

**Fig. 1.**
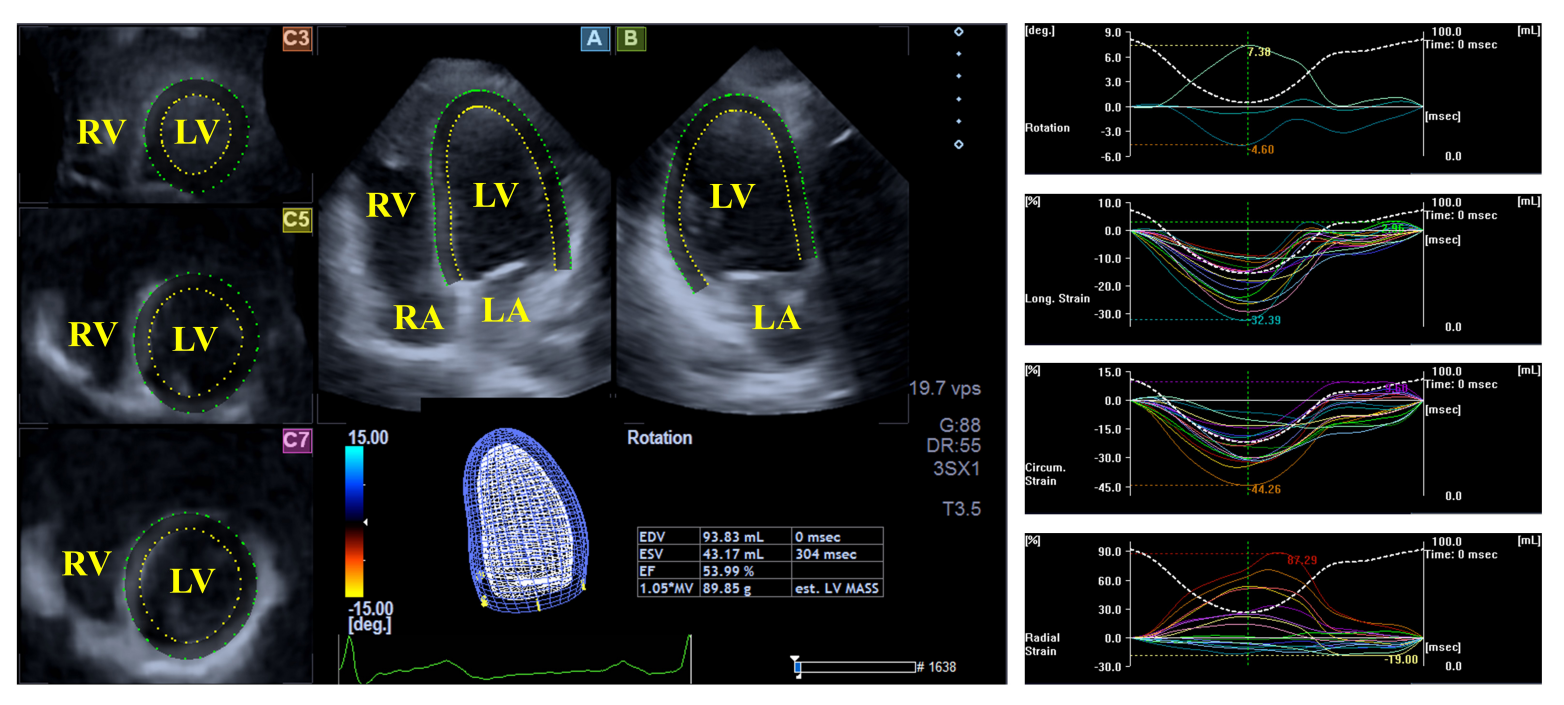
**Evaluation of the left ventricular volumes, rotational parameters and strains by three-dimensional speckle-tracking echocardiography**. Abbreviations: LA, left atrium; RA, right atrium; LV, left ventricle; RV, right ventricle.

The left ventricle (LV) exhibits a bullet or egg-like shape; the LV fills from the left atrium (LA) through the mitral apparatus and empties into the aorta through the aortic valve [22, 23]. Myocardial architecture of the LV is special; the LV includes left-handed directed subepicardial fibers, with circumferentially running mid-layer fibers, and right-handed directed subendocardial fibers. This structure allows not only a radial, circumferential and longitudinal deformation of the LV represented by unidimensional/unidirectional radial, longitudinal and circumferential strains, but also a rotation of the oppositely directed basal and apical LV regions, namely in clockwise and counterclockwise directions, respectively, during systole, creating a LV twist, the movement of which under healthy circumstances is similar to that of wringing a towel. In diastole, LV untwisting is seen, which is the opposite of the movement observed in systole, resulting in lower end-systolic and larger end-diastolic volumes (Fig. 1) [22, 23, 24].

#### 3.1.2 In Hemophilia

Increased septal thickness was demonstrated in hemophilia patients in an early echocardiographic study. Doppler imaging confirmed that 18% of hemophilia patients had diastolic dysfunction with an impaired relaxation pattern, and another 20% had a restrictive filling pattern [25]. Moreover, late diastolic velocity of the septum, systolic velocity of the lateral mitral valve (MV), late diastolic velocity of the lateral mitral annulus (MA), and late velocity of the tricuspid annulus (TA) measured by tissue Doppler echocardiography differed significantly compared to the control group [25]. Increased myocardial performance index, indicating deteriorated LV myocardial systolic function, was demonstrated in hemophilia A patients and was enhanced with increasing arterial stiffness [26].

Based on the findings from the MAGYAR-Path Study, no differences between mean segmental and global LV strains, as assessed by 3DSTE, could be demonstrated in hemophilia cases as compared to those of controls. LV circumferential strains of the basal and midventricular regions were impaired in hemophilia patients, suggesting subclinical LV regional dysfunction [18]. Moreover, reduced apical LV rotation and twist in degrees were present in adult individuals with hemophilia; the prevalence of the absence of LV rotational mechanics, called ‘rigid body rotation’ of the LV, was similar to that of healthy subjects (7%) [19].

### 3.2 Left Atrium

#### 3.2.1 In Healthy Subjects 

**Fig. 2.**
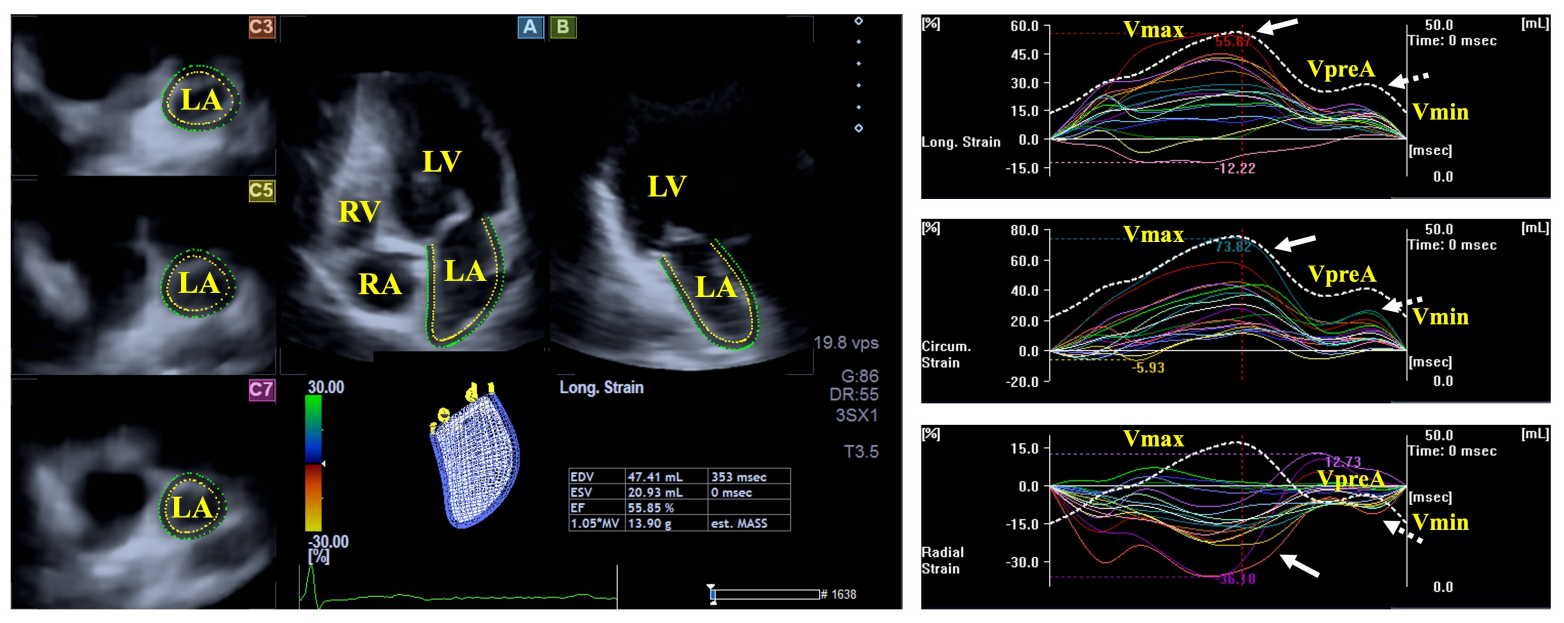
**Evaluation of the left atrial volumes and strains by three-dimensional speckle-tracking echocardiography**. Abbreviations: LA, left atrium; RA, right atrium; LV, left ventricle; RV, right ventricle; Vmin, end-diastolic minimum volume of the LA; VpreA, early diastolic pre-atrial contraction volume of the LA; Vmax, maximum end-systolic volume of the LA, white arrow represents LA reservoir strain, dashed white arrow represents LA strain at atrial contraction.

The LA fills from the four pulmonary veins and empties into the LV via the mitral apparatus. The muscular structure of the LA differs significantly from that of the LV, with bands in both the longitudinal (e.g., parietal septoatrial band) and circumferential (e.g., basal interatrial band) directions. The main LA muscles are attached to the rim of the oval fossa, providing mechanical support. During the cardiac cycle, the LA has three main functions: a systolic reservoir (promotes the largest LA volume), an early diastolic conduit (volume of the LA before atrial contraction), and a late diastolic booster pump function (actively contracting chamber with the smallest LA volume). Emptying fractions, stroke volumes, and strains can serve as characteristics of the LA function representing all its phases (Fig. 2) [27, 28].

#### 3.2.2 In Hemophilia 

Results from the MAGYAR-Path Study suggest that volumetric abnormalities of the LA could not be demonstrated in hemophilia patients. While the total atrial emptying fraction was found to be impaired, peak mean segmental circumferential and longitudinal LA strains were also deteriorated in individuals with hemophilia compared to controls featuring LA reservoir function. All other derived volume-based functional features and strains did not differ between hemophilia patients and controls [20].

### 3.3 Mitral Valve

#### 3.3.1 In Healthy Subjects 

**Fig. 3.**
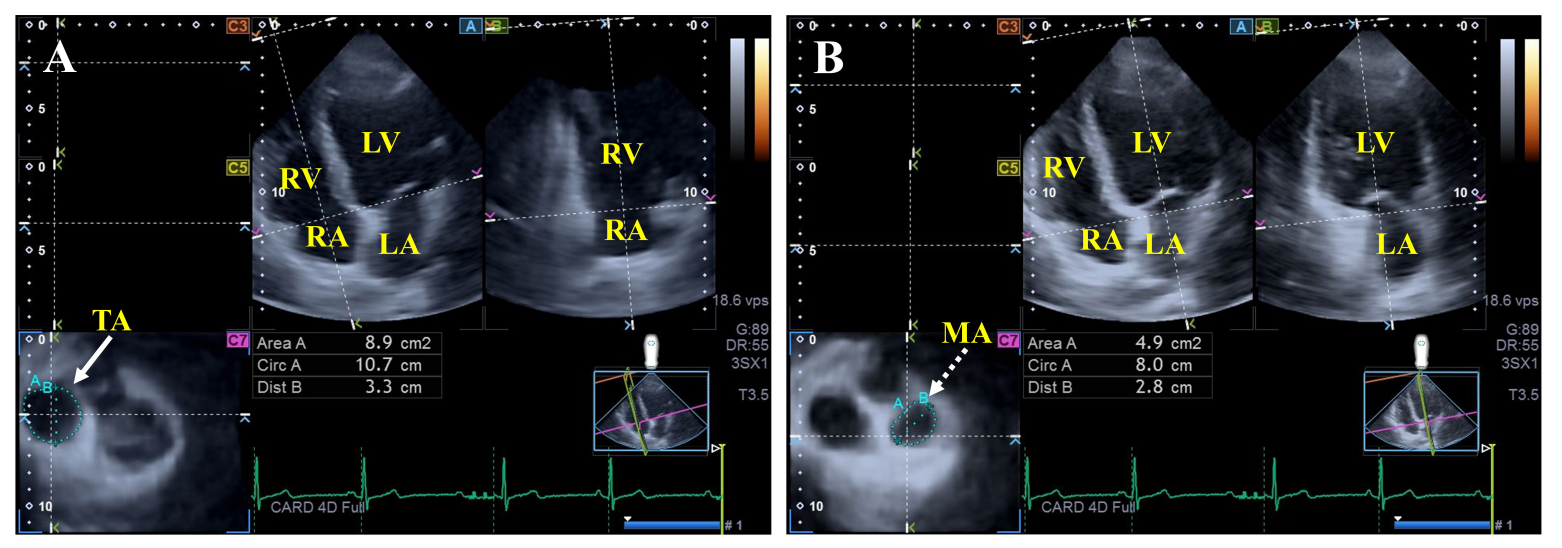
**Assessment of the tricuspid (A) and mitral annular (B) dimensions by three-dimensional speckle-tracking echocardiography**. Abbreviations: LV, left ventricle; RA, right atrium; LA, left atrium; RV, right ventricle; Dist, diameter; Area, area; Circ, perimeter; TA, tricuspid annulus; MA, mitral annulus.

The MV has a hyperbolic paraboloid saddle-shape located between the LA and LV with a 3D dynamic movement during the heart cycle. The MV is composed of the tendineal chords with papillary muscles, the posterior and the anterior leaflets, and the MA. The proper movement of the mitral apparatus is provided by the coordinated contraction of adjacent LV and LA areas (Fig. 3) [29, 30].

#### 3.3.2 In Hemophilia 

Findings from the MAGYAR-Path Study showed dilated MA diameters, areas, and perimeters measured both in end-systole and end-diastole in hemophilia patients, together with consequentially impaired MA fractional area change compared to healthy controls. Moreover, mitral valve abnormalities could not be detected in this study with hemophilia patients without any suspicion of cardiovascular pathologies [21].

### 3.4 Aortic Valve

#### 3.4.1 In Healthy Subjects 

The aortic valve (AV) is the outlet of the LV, in addition to being a gateway to systemic circulation. In normal circumstances, the AV has three thin semilunar leaflets that allow blood to flow in one direction in systole without allowing backflow during diastole [31].

#### 3.4.2 In Hemophilia

In a small study of hemophilia patients, none of the cases exhibited aortic valve incompetence larger than/equal to grade 1 or significant stenosis [21].

### 3.5 Aorta

#### 3.5.1 In Healthy Subjects 

The aorta is the main artery, an elastic tissue, which acts not only as a conduit, but is a reservoir via the Windkessel effect, allowing temporary storage of blood in systole. Increased aortic stiffness is a maladaptive response to hemodynamic forces, a measure of wall elasticity, which has a significant prognostic impact affecting blood pressure, LV relaxation, coronary perfusion, etc. [32, 33].

#### 3.5.2 In Hemophilia

Increased arterial stiffness in hemophilia A patients was found to be associated with the systolic function of the LV [26]. However, in another recent study, arterial stiffness did not differ between controls and hemophilia patients, which could be partly explained by the age (children vs. middle-aged adults), differences in the activity of the disease, selection of the control subjects, etc. [7].

## 4. The Right Heart and the Pulmonary Artery

### 4.1 Right Ventricle

#### 4.1.1 In Healthy Subjects 

The right ventricle (RV) empties into the pulmonary artery through the pulmonary valve. In contrast, the RV fills from the right atrium (RA) through the tricuspid valve (TV). From the front, the RV is located around the LV; the RV has a triangular shape, a cross-sectional appearance that is crescent-like, and the diameter increases from the apex to the base. The wall thickness of the RV is approximately 3–5 mm, less than that of the LV, but the apical part is more trabeculated than that of the LV. The muscular architecture of the RV is special, with a free wall containing superficial fibers of the subepicardium arranged in a transverse direction, showing a bellows effect via radial RV free wall movement, while deep, longitudinally arranged subendocardial fibers extend from the base towards the apex. The superficial muscle fibers of the RV continue onto the LV, contributing to ventricular interdependence [34, 35, 36, 37].

#### 4.1.2 In Hemophilia 

There is limited information regarding the RV in hemophilia. However, from Doppler parameters, peak TV velocity measured at late diastole (A) was found to be reduced with preserved early peak velocity (E), resulting in larger E/A in hemophilia patients compared to controls, which is suggestive of mild RV alterations. From tissue Doppler parameters, early and late diastolic and systolic wave velocities of the tricuspid valve were similar between hemophilia individuals and controls [25].

### 4.2 Right Atrium

#### 4.2.1 In Healthy Subjects 

The blood from the RA leaves into the RV through the TV and fills from the coronary sinus and the caval veins. The RA consists of the vestibule, the auricle, and the venous part. Moreover, the muscle fibers run in longitudinal and circumferential directions in the RA. The terminal crest and the terminal pectinate muscles are the most important muscles in the RA. The triple function of the RA is similar to that of the LA, having a systolic reservoir phase with a maximal RA volume, an early diastolic conduit phase with an RA volume called “pre-atrial contraction”, and a late diastolic active contraction with a minimal RA volume. The sinus node, the baroreceptors, and the production of natriuretic peptides are also integral parts and functions of the RA [38].

#### 4.2.2 In Hemophilia 

Currently, no study is available regarding RA morphology and functional characteristics in hemophilia.

### 4.3 Tricuspid Valve

#### 4.3.1 In Healthy Subjects

The TV is located between the RV and RA, presenting a saddle-shaped 3D morphology with an asymmetrical ellipsoid annulus, anterior, posterior, and septal leaflets with papillary muscles and tendineal chords [39].

#### 4.3.2 In Hemophilia 

Preserved end-systolic TA sizes and dilated end-diastolic TA perimeter, area, and diameter were present together with reduced TA fractional area change in hemophilia [21]. No data in the literature support that other TV abnormalities are more frequently associated with hemophilia patients compared to controls.

### 4.4 Pulmonary Valve

#### 4.4.1 In Healthy Subjects 

The pulmonary valve is the gateway of the pulmonary circulation and the outlet of the RV with three semilunar leaflets [40].

#### 4.4.2 In Hemophilia 

Literature data do not support that pulmonary valve pathologies occur at different rates compared to controls [21].

### 4.5 Pulmonary Artery

#### 4.5.1 In Healthy Subjects 

The pulmonary artery is the largest artery involved in the pulmonary circulation [37].

#### 4.5.2 In Hemophilia 

No current literature data support that the pulmonary artery shows any pathology in hemophilia as compared to controls [21].

## 5. Pathophysiological Background

Classic cardiovascular risk factors such as hypertension are prevalent in hemophilia, which could partially explain these findings, together with those associated with aging. Various factors could explain abnormalities in regional LV deformation in hemophilia, including increased aortic stiffness; however, the prevalence of these abnormalities in hemophilia remains unclear. Theoretically, the changes in blood quality caused by hemophilia and the resulting alterations, including a hypocoagulable state, could be partly responsible for the abnormalities presented above; however, further studies are required to confirm this theory.

## 6. Clinical Implications

Although hemophilia is a hematological disease resulting from the reduced production of coagulation factors, the disease is accompanied by some abnormalities in the morphology and function of the cardiac chambers and valves. Although these abnormalities are mild and do not seem to have an essential impact on the outcome of the pathology, further investigations are warranted to perform improved assessments. Indeed, modern cardiovascular imaging procedures are now available and may help to perform enhanced assessments.

## 7. Conclusions

Hemophilia is a hematological disorder resulting from the lack of coagulation factor formation. Although limited and unclear, sometimes contradictory data are available on cardiovascular morphology and functional properties in hemophilia (Table 1, Ref. [18, 19, 20, 21, 25, 26]). Overall, it can be concluded that abnormalities can be observed in regional LV myocardial function and LV rotational mechanics, in LA reservoir function without volumetric changes, and in the dimensions and functional properties of the MA and TA. Some single studies also suggest mild abnormalities in the RV. In some cases, these abnormalities can be explained by the disease itself and its pathophysiological basis; however, a relationship can also be assumed with other disease, such as classic risk factors.

**Table 1.** **Summarization of the most important findings**.

		Reference
LEFT HEART		
Left ventricle	In total, 18% of hemophilia patients had diastolic LV dysfunction with an impaired relaxation pattern, and another 20% had a restrictive filling pattern	[25]
	Late diastolic velocity of the septum, systolic velocity of the lateral MV, late diastolic velocity of the lateral MA, and late velocity of the TA differed significantly between hemophiliacs and controls	[25]
	Myocardial performance index is increased, suggesting LV systolic dysfunction	[26]
	No differences between mean segmental and global LV strains were observed between hemophilia cases and controls. Basal and midventricular LV-CS were impaired in hemophilia patients	[18]*
	Reduced apical LV rotation and twist are present in hemophilia patients	[19]*
	The prevalence of LV-RBR was similar to that for healthy subjects	[19]*
Left atrium	LA volumetric abnormalities in hemophilia were not present. Some volume-based LA functional properties featuring LA reservoir function were impaired	[20]*
Mitral valve	MA dimensions were dilated in hemophilia patients, together with consequentially impaired MA fractional area change	[21]*
Aorta	Increased arterial stiffness in hemophilia A was found to be associated with systolic function of the LV	[26]
RIGHT HEART		
Right ventricle	From Doppler parameters, peak TV velocity measured at late diastole (A) was reduced with preserved early peak velocity (E), resulting in a larger E/A. From tissue Doppler parameters, early and late diastolic and systolic wave velocity of the TV were similar between hemophilic individuals and controls	[25]
Tricuspid valve	End-systolic TA sizes were preserved with dilated end-diastolic TA dimensions and reduced TA fractional area change in hemophilia patients	[21]*

Abbreviations: CS, circumferential strain; LA, left atrial; LV, left ventricular; LV-RBR, left ventricular ‘rigid body rotation’; MA, mitral annulus; MV, mitral valve; TV, tricuspid valve. The star (*) represents studies from the MAGYAR-Path Study.
